# UltraAIGenomics: Artificial Intelligence-Based Cardiovascular Disease Risk Assessment by Fusion of Ultrasound-Based Radiomics and Genomics Features for Preventive, Personalized and Precision Medicine: A Narrative Review

**DOI:** 10.31083/j.rcm2505184

**Published:** 2024-05-22

**Authors:** Luca Saba, Mahesh Maindarkar, Amer M. Johri, Laura Mantella, John R. Laird, Narendra N. Khanna, Kosmas I. Paraskevas, Zoltan Ruzsa, Manudeep K. Kalra, Jose Fernandes E Fernandes, Seemant Chaturvedi, Andrew Nicolaides, Vijay Rathore, Narpinder Singh, Esma R. Isenovic, Vijay Viswanathan, Mostafa M. Fouda, Jasjit S. Suri

**Affiliations:** ^1^Department of Radiology, Azienda Ospedaliero Universitaria, 40138 Cagliari, Italy; ^2^School of Bioengineering Sciences and Research, MIT Art, Design and Technology University, 412021 Pune, India; ^3^Stroke Monitoring and Diagnostic Division, AtheroPoint™, Roseville, CA 95661, USA; ^4^Department of Medicine, Division of Cardiology, Queen’s University, Kingston, ON K7L 3N6, Canada; ^5^Department of Medicine, Division of Cardiology, University of Toronto, Toronto, ON M5S 1A1, Canada; ^6^Heart and Vascular Institute, Adventist Health St. Helena, St Helena, CA 94574, USA; ^7^Department of Cardiology, Indraprastha APOLLO Hospitals, 110001 New Delhi, India; ^8^Department of Vascular Surgery, Central Clinic of Athens, 106 80 Athens, Greece; ^9^Invasive Cardiology Division, University of Szeged, 6720 Szeged, Hungary; ^10^Department of Radiology, Harvard Medical School, Boston, MA 02115, USA; ^11^Department of Vascular Surgery, University of Lisbon, 1649-004 Lisbon, Portugal; ^12^Department of Neurology & Stroke Program, University of Maryland, Baltimore, MD 20742, USA; ^13^Vascular Screening and Diagnostic Centre and University of Nicosia Medical School, 2368 Agios Dometios, Cyprus; ^14^Nephrology Department, Kaiser Permanente, Sacramento, CA 95823, USA; ^15^Department of Food Science and Technology, Graphic Era Deemed to be University, Dehradun, 248002 Uttarakhand, India; ^16^Department of Radiobiology and Molecular Genetics, National Institute of The Republic of Serbia, University of Belgrade, 11000 Belgrade, Serbia; ^17^MV Diabetes Centre, Royapuram, 600013 Chennai, Tamil Nadu, India; ^18^Department of Electrical and Computer Engineering, Idaho State University, Pocatello, ID 83209, USA; ^19^Department of Computer Engineering, Graphic Era Deemed to be University, Dehradun, 248002 Uttarakhand, India

**Keywords:** cardiovascular disease, stroke, radiomics, genomics, artificial intelligence, deep learning, bias, pruning, explainable AI

## Abstract

Cardiovascular disease (CVD) diagnosis and treatment are challenging since symptoms appear late in the disease’s progression. Despite clinical risk scores, cardiac event prediction is inadequate, and many at-risk patients are not adequately categorised by conventional risk factors alone. Integrating genomic-based biomarkers (GBBM), specifically those found in plasma and/or serum samples, along with novel non-invasive radiomic-based biomarkers (RBBM) such as plaque area and plaque burden can improve the overall specificity of CVD risk. This review proposes two hypotheses: (i) RBBM and GBBM biomarkers have a strong correlation and can be used to detect the severity of CVD and stroke precisely, and (ii) introduces a proposed artificial intelligence (AI)—based preventive, precision, and personalized (${\mathrm{aiP}}^{3}$) CVD/Stroke risk model. The PRISMA search selected 246 studies for the CVD/Stroke risk. It showed that using the RBBM and GBBM biomarkers, deep learning (DL) modelscould be used for CVD/Stroke risk stratification in the ${\mathrm{aiP}}^{3}$ framework. Furthermore, we present a concise overview of platelet function, complete blood count (CBC), and diagnostic methods. As part of the AI paradigm, we discuss explainability, pruning, bias, and benchmarking against previous studies and their potential impacts. The review proposes the integration of RBBM and GBBM, an innovative solution streamlined in the DL paradigm for predicting CVD/Stroke risk in the ${\mathrm{aiP}}^{3}$ framework. The combination of RBBM and GBBM introduces a powerful CVD/Stroke risk assessment paradigm. ${\mathrm{aiP}}^{3}$ model signifies a promising advancement in CVD/Stroke risk assessment.

## 1. Introduction

Cardiovascular diseases (CVD) are the leading cause of death worldwide, contributing to 17.3 million deaths annually and an estimated 23.6 million by 2030 [1, 2]. CVD will cost $920 billion in direct medical expenses by 2030, thus making it a critical concern for the public health system [3]. Coronary heart disease (CHD) is the leading cause of CVD, with atherosclerosis, a chronic inflammatory condition of the artery wall, among the most common causes of death [4, 5]. CVD is correlated with genetic, metabolomic, environmental, behavioural, and lifestyle characteristics [6, 7]. The most utilized techniques for predicting CVD risk are based solely on traditional risk factors like age, gender, high cholesterol, high blood pressure, smoking, and comorbidities such as diabetes mellitus [8] and hypertension [9, 10]. This is because laboratory-based biomarkers are costly and impossible in developing countries with “resource constraints” [11].

Most CVD risk-scoring measurement systems were designed for Caucasians [12, 13]. Meanwhile, various ethnicities, such as South Asian and Indian, were not considered during the development of these systems [14]. Due to this error, there may be misdiagnoses and suboptimal treatment outcomes, raising questions regarding the generalizability and validity of these models for non-cohort data. Hence, the validity and usefulness of all these prediction models in groups apart from white cohorts remain unknown, which is a substantial constraint in the current research [9]. It results in complex diagnoses and treatments to under-estimation or over-estimation, the so-called misdiagnosis of CVD risk [15, 16]. As a result, there is an obvious need to resolve the poorly managed misdiagnosis challenges [17].

Moreover, the relationship between traditional risk factors and CVD outcomes is often assumed to be linear. However, when we consider factors like ethnicity and genetic predispositions, this relationship becomes more complex and non-linear. The non-linear risk stratification is better using artificial intelligence (AI) as it understands the critical points and accordingly customizes the risk predictions, enhancing the granularity and accuracy of CVD risk assessment models.

**Fig. 1.**
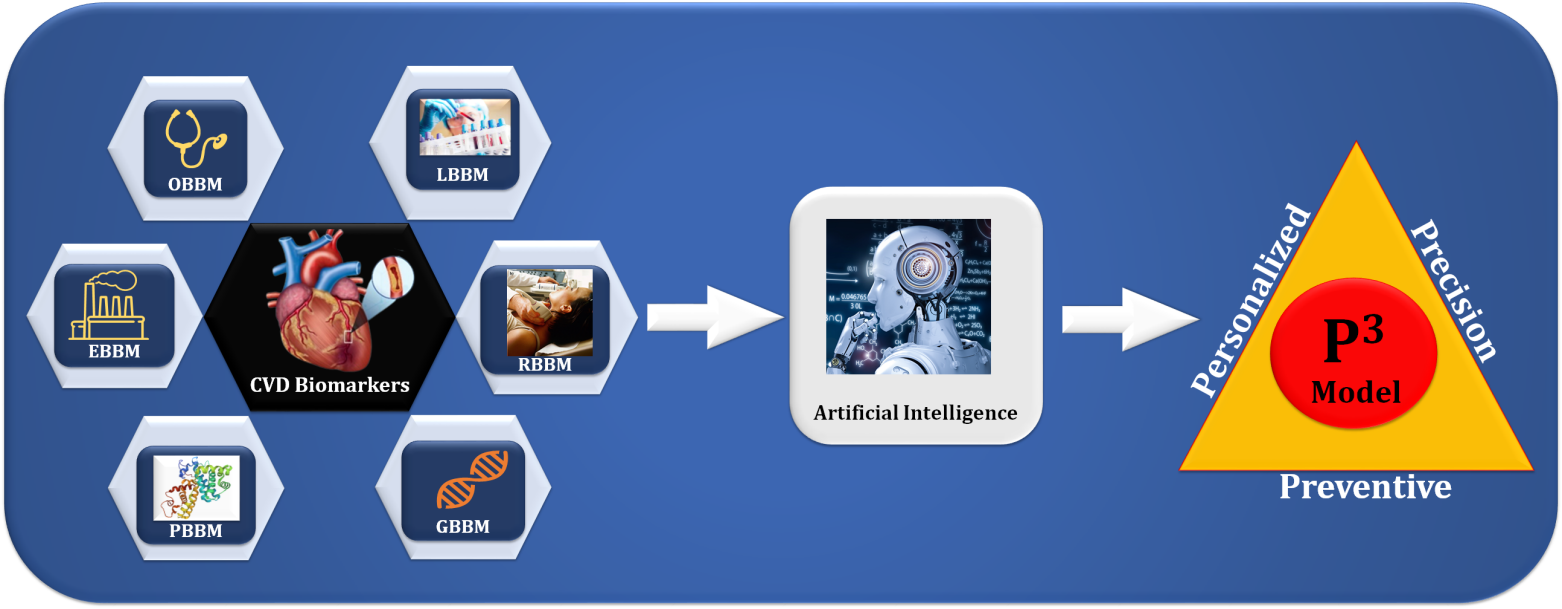
**The overview of composite biomarkers using an AI model for the preventive, personalized, and precise (${\mathrm{aiP}}^{3}$) solution leads to multiclass CVD risk assessment**. CVD, cardiovascular disease; OBBM, office-based biomarkers; LBBM, laboratory-based biomarkers; RBBM, radiomics-based biomarkers; GBBM, genomics-based biomarkers; PBBM, proteomics-based biomarkers; EBBM, environment-based biomarkers.

Most recently, a paradigm shift has occurred towards precision medicine, and the use of AI, in particular, has emerged as a viable solution to these problems [18]. Former USA President Obama introduced the precision medicine initiative (PMI) in his 2015 State of the Union speech [19, 20]. PMI will be a “milestone” initiative (if funded) that presents a unique potential for scientists and clinicians to mobilize collective resources and expertise to develop and spread the knowledge needed to translate discoveries to reduce the worldwide burden of CVD [21]. The precision medicine approach, with the help of AI, can improve symptom-driven care by proactively combining multi-omics assessments with clinical [22, 23], imaging [24, 25, 26], epidemiological [27, 28], and demographic variables [29]. Precision medicine allows for earlier treatments for advanced diagnostics and tailoring better and more affordable personal treatment [30, 31, 32]. The concept of precision medicine is centred on the predictive, preventive, and personalized (**$P^{3}$**) approach for the 360-degree care of the patient. Fig. 1 shows an integration of various CVD biomarkers, namely office-based biomarkers (OBBM), laboratory-based biomarkers (LBBM), radiomics-based biomarkers (RBBM), genomics-based biomarkers (GBBM), proteomics-based biomarkers (PBBM), and environment-based biomarkers (EBBM) feed to the AI model for the CVD/Stroke risk stratification in the **$P^{3}$** environment [32]. Each patient attempts to help clinicians understand how personalized medical information variations might contribute to health and effectively diagnose and anticipate the most effective approach for a patient’s treatment [33].

We propose in this study a novel method using deep learning (DL) to risk stratify the CVD/Stroke that combines RBBM and GBBM as covariates. Furthermore, due to difficulties such as a lack of clinical assessment and validation and imbalanced data sets, DL algorithms, particularly DL-based prediction systems, can exhibit bias and lack generalization. Therefore, we discuss the potential solutions to these challenges [34, 35]. As the importance of reducing the size of DL-based prediction systems for miniature medical devices such as edge devices, we investigated pruned or compacted AI systems for CVD risk using multi-omics data [36]. Finally, we use the explainability model [37] to illuminate AI’s “Black Box Nature” and, lastly, to implement such paradigms into a cloud-based framework [38, 39]. This presented study aims to analyze DL systems for CVD risk stratification using the UltraAIGenomics model by AtheroPoint™ (Roseville, CA, USA) with the goals of the ${\mathrm{aiP}}^{3}$, reducing bias, increasing compression, and making the results clinically explainable in a cloud/telemedicine setting.

This review examines recent CVD risk assessment advancements, focusing on integrating AI and precision medicine. The key contributions of the paper are:

∙ Ethnic Diversity Integration: To address the underrepresentation of ethnic diversity in current CVD risk models, this paper proposes an AI-powered framework that considers various demographic factors, including genetics and environment.

∙ Non-Linear Risk Stratification: This approach improves accuracy in assessing non-linear risk across diverse populations using sophisticated deep learning algorithms.

∙ AI-driven Customization: This approach uses AI to comprehend important data points and personalize risk assessments, providing accurate and flexible risk evaluations for a range of patient profiles.

∙ Explainable AI Framework: Examines how to apply explainable AI frameworks to improve clinician confidence and speed up the uptake of AI-driven models for CVD risk assessment.

The structure of this study can be outlined as follows: Section 2 introduces the search strategy and presents the statistical distribution. Section 3 delves into radiomics-based biomarkers as integral components for AI-powered CVD diagnosis. Section 4 focuses on genomics-based biomarkers, which are key features for AI-based CVD diagnosis. In Section 5, we explore the role of UltraAIGenomics and the implementation of ${\mathrm{aiP}}^{3}$-based DL for CVD risk stratification. Section 6 comprehensively discusses factors impacting CVD, including explainability, pruning, blockchain integration, and other miscellaneous factors. The progressive growth of CVD risk calculators from conventional to AI-based and its practical implications are presented in Section 7. Section 8 delves into critical discussions about DL models. Lastly, Section 9 concludes the presented study.

## 2. Search Strategy and Statistical Distribution

**Fig. 2.**
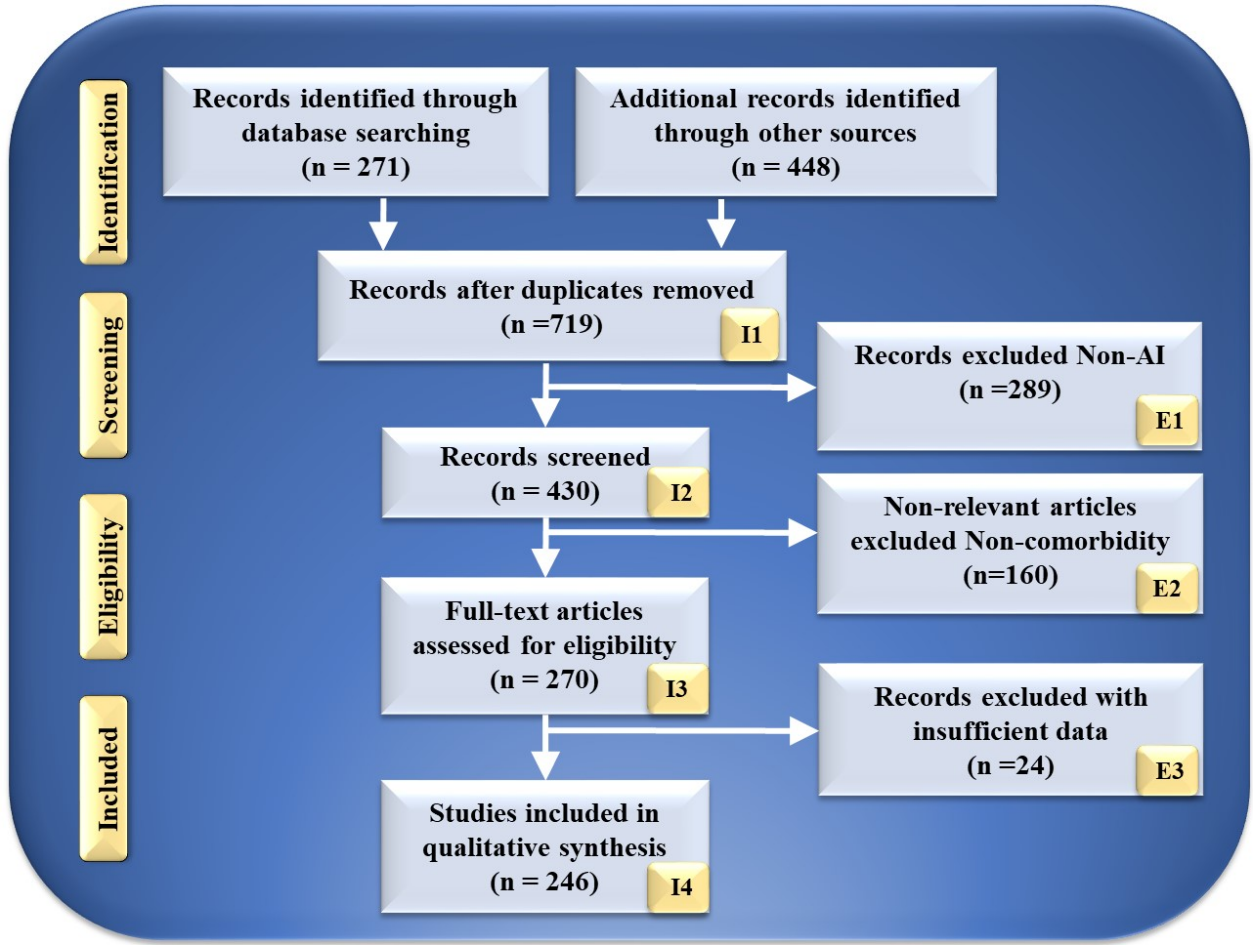
**PRISMA model for study selection**. I, included; E, excluded; AI, artificial intelligence.

The search strategy utilized by the PRISMA paradigm is depicted in Fig. 2. Using keywords such as “cardiovascular disease”, “stroke”, “CVD”, “genomics and CVD”, “radiomics and CVD”, “radiomics and stroke”, “genomics and stroke”, “prevention medicine”, “preventive medicine and CVD”, “personalized medicine and artificial intelligence”, “atherosclerotic in genomics”, “radiomics and AI”, “genomics and AI”, and “artificial intelligence”. PubMed and Google Scholar were used to identify and screen relevant papers. There was a total of 271 entries in the database search, and there was a total of 448 items from other sources. After using quality-specific parameters such as timeliness and relevance, this number was decreased to 719 articles.

This review considered 430 publications in total. The three criteria for elimination were: (i) unrelated research; (ii) irrelevant papers; and (iii) inadequate data. This resulted in the exclusion of 289, 160, and 34 studies, as indicated by E1, E2, and E3, resulting in the final assessment of 246 studies. These studies lack AI description or do not demonstrate risk categorization for CVD or stroke in RBBM and GBBM. Following the PRISMA methodology, 289 studies were eliminated from the screening process and designated E1. Only irrelevant research is excluded from the CVD/Stroke area of view. They are not addressed in RBBM, GBBM, CVD, and stroke. In this investigation, we are interested in articles linking CVD/Stroke with RBBM and GBBM. If the research indicated a correlation between Parkinson’s disease, cancer, and diabetes, the study was not considered. This category had 160 studies, as indicated by E2 in the PRISMA model. These studies lacked sufficient information to be included in our analysis or failed to demonstrate a connection between RBBM, GBBM, and CVD/Stroke. Such conversations were not pursued because neither RBBM nor CVD risk factors, such as LBBM, were considered. In addition, they lacked adequate selectable AI and CVD/Stroke characteristics for analysis that could be utilized for CVD/Stroke risk stratification. This AI algorithm may be a hybrid deep learning (HDL) or neural network (NN) for CVD/Stroke risk classification. We found 24 research studies with inadequate data sets designated as E3 in the PRIMSA model. We then performed a narrative synthesis of the data, depending on the nature and quality of the included studies.

## 3. Radiomics-Based Biomarkers as Features for AI-Based CVD Diagnosis 

Biomarkers are important in both disease diagnosis and the development of drugs for the treatment of diseases. Biomarkers can be categorized as prognostic, pharmacodynamic, or predictive from the perspective of precision medicine [40]. This section discusses the CVD biomarkers (OBBM, LBBM, RBBM, and GBBM) utilized as AI features for CVD risk assessment. Currently, the evaluation of CVD risk factors such as age, gender, baseline systolic and diastolic blood pressure levels, serum cholesterol, smoking status, and diabetes history is conventionally required to predict a patient’s CVD/Stroke risk over one to 10 years or a life-long period. In recent years, various radiological methods have been invented and widely used to rule out and/or identify preclinical atherosclerotic-based CVD to advise optimal prophylactic therapy. Since the carotid artery can be used for the prediction of coronary artery disease [41, 42, 43, 44], the most commonly used imaging modalities for its screening are magnetic resonance imaging (MRI) [45, 46, 47], computed tomography angiography (CTA) [48, 49, 50, 51, 52], optical coherence tomography (OCT) [53], and ultrasound (US) [54, 55]. However, the US is the most common, user-friendly, cost-effective, high-resolution, non-invasive image acquisition modality capable of imaging and recognizing atherosclerotic plaque [54, 56, 57]. Therefore, it offers a wide range of applications for regular proactive monitoring of atherosclerotic plaque for CVD risk assessment [58, 59, 60, 61, 62, 63].

As shown in Table 1 (Ref. [41, 64, 65, 66, 67, 68, 69, 70, 71, 72, 73]), the studies use stochastics-based methods (SBM) to stratify the CVD risk. Delsanto *et al. * [64] proposed a CULEX algorithm for the feature extraction of carotid intima-media thickness (cIMT) and wall thickness (cWT). The typical margin of error for cIMT estimations was 7%. This performance was comparable to the gold standard reading. These techniques yielded accuracies between 88.07% to 98.06%. Most SBM studies use segmentation and multiresolution-based scale-space methods for segmentation [41, 65, 74, 75, 76, 77, 78]. The scale-space-based methods were used to extract the image-based phenotypes, mainly plaque burden, plaque area (PA), carotid intima-media thickness (cIMT), intima-media thickness variability (IMTV), stenosis, and lumen diameter (LD) and its variations [41, 65, 66, 67, 68, 69]. Other studies used Spearman’s [70], Shapiro-Wilk [71], and Kaplan-Meier’s [72]statistical-based methods for the estimation of cIMT, IMTV, and LD. Table 2 (Ref. [79, 80, 81, 82, 83, 84, 85, 86, 87, 88]) shows the studies that used DL-based radiomics (covariates) to segment carotid B-mode ultrasound (cBUS). Most of the studies used UNet [79, 80, 81, 82, 89, 90], UNet++ [83], and convolution neural network (CNN) [84] as classifiers and segmentation for the cIMT region in carotid scans.

**Table 1.** **Studies using non-AI-based (SBM) radiomics for segmentation and quantification (features) using cBUS**.

SN	Studies (Author and Citation)	Year	DS	Artery Segment (CCA/ICA/CB)	IM	Method (Algorithm)	Feature (Covariates)	Performance (ACC, *p*-value)	Conclusion (Relationships)
1	Delsanto *et al*. [64]	2007	120	CCA	US	CULEX	cIMT and cWT	Error <7%	cIMT and Plaque ROI extraction.
2	Molinari *et al*. [66]	2010	200	CCA	US	Scale-space	cIMT, IMTV	ACC: 88.90%	Tissue characterization of plaque.
3	Ikeda *et al*. [73]	2013	218	CCA	US	Threshold	cIMT	ACC: 90.5%	cIMT and Plaque ROI extraction and segmentation.
4	Araki *et al*. [41]	2014	100	CCA	IVUS	Scale-space	LD, PA	ACC: 91.04%	cIMT (R) vs. CCA > cIMT (L) vs. CCA
5	Ikeda *et al*. [65]	2017	370	CCA	US	Scale-space	cIMT	ACC: 88.07%, AUC: 0.91 (*p* < 0.0001)	PA in Bulb > PA (CCA)
6	Acharya *et al*. [67]	2013	404	CCA	US	Scale-space	cIMT, LD	ACC: 98.70%	High plaque volume narrowing PA LD/IAD.
7	Ikeda *et al*. [68]	2015	649	CCA	US	Scale-space	cIMT	ACC: 98.86%	PA in Bulb > PA (CCA)
8	Saedi *et al*. [69]	2018	100	CCA	US	Scale-space	cIMT, LD	SYNTAX score 15.76 + 4.82	SYNTAX score and cIMT have no relation.
9	Lucatelli *et al*. [70]	2016	122	ICA	US	Spearman’s	LA, LD	ACC: 88.05%, AUC: 0.91 (*p* < 0.0001)	IMTV has a strong relationship with LA volume.
10	Cloutier *et al*. [71]	2018	6101	CCA	US	Shapiro-Wilk	PA, and cIMT	(chi-square 450, *p* < 0.0001) > (chi-square 450, *p* < 0.0001)	A carotid plaque has a stronger relation with CAC.
11	Johri *et al*. [72]	2021	514	CCA	US	Kaplan-Meier	MPH	CI = 0.99–2.4, *p* = 0.06	MPH quantification of CCA helps to predict CVD.

AI, artificial intelligence; cBUS, carotid B-mode ultrasound; SN, serial number; DS, data size; IM, imaging modality; IVUS, intra-vascular ultrasound; US,ultrasound; SBM, stochastics-based methods; cWT, carotid wall thickness; cIMT, carotid intima-media thickness; LD, lumen diameter; CVD, cardiovascular disease; PA, plaque area; CCA, common carotid artery; IMTV, intima media thickness variability; CI, confidence interval; CB, carotid bifurcation; CAC, coronary artery calcium; MPH, maximum plaque height; ICA, internal carotid artery; ACC, accuracy; AUC, area under the curve; ROI, region of interest; LA, left atrium.

**Table 2.** **Studies using DL-based radiomics (covariates) for segmented features using cBUS**.

SN	Studies	Year	DS	Artery Segment	IM	AI (ML/DL)	Classifier Type	Segment Features	Performance	Conclusion
1	Saba *et al*. [85]	2018	100	CCA	US	ML	SVM, RF	LD	ACC: 98.32%	Intra/inter-observer variability.
2	Biswas *et al.* [86]	2020	250	CCA	US	DL	CNN, LR	cWT, PB	cIMT error <0.093 ± 0.06 77 mm, AUC: 0.89 (*p* < 0.0001)	Joint detection cWT and PB.
3	Vila *et al.* [87]	2020	8000	CCA	US	DL	CNN (Dense Net)	cIMT	ACC: 96.45%, AUC: 0.89 (*p* < 0.0001)	Plaque detection and cIMT estimation.
4	Jain *et al*. [79]	2021	970	CCA	US	DL	UNet, UNet+	PA	ACC: 88%, AUC: 0.91 (*p* < 0.0001)	Detection of PA and segmentation.
5	Jain *et al.* [83]	2022	379	ICA	US	DL	UNet, UNet+	PA	AUC: 97%, AUC: 0.99 (*p* < 0.0001)	Detection of PA and segmentation.
6	Yuan *et al.* [80]	2022	115	CCA	US	DL	UNet	cIMT	ACC: 97%, Dice 83.3–85.7	cIMT and plaque segmentation.
7	Molinari *et al.* [84]	2012	500	CCA	US	DL	CNN	cIMT and cWT	ACC: 95.6%, AUC:0.83 (*p* < 0.0001)	cIMT and cWT measurement.
8	Gago *et al.* [81]	2022	8000	CCA	US	DL	UNet	PA, cIMT, and cWT estimation	ACC: 79.00%	Tissue characterization of plaque.
9	Shin *et al.* [88]	2022	1440	CCA	US	DL	CNN	Plaque viscous index	ACC: 83.00%, AUC: 0.87 (*p* < 0.0001)	Viscoelasticity index.
10	Lainé *et al.* [82]	2022	2676	CCA	US	DL	UNet	cWT	ACC: 86.00%	Dilated U-net architecture is used for cWT.

cBUS, carotid B-mode ultrasound; SN, serial number; DS, data size; IM, imaging modality; ICA, internal carotid artery; US, ultrasound; cWT, carotid wall thickness; cIMT, carotid intima-media thickness; LD, lumen diameter; PB, plaque burden; PA, plaque area; CCA, common carotid artery; ICA, internal carotid artery; ACC, accuracy; AUC, area under the curve; AI, artificial intelligence; ML, machine learning; DL, deep learning; SVM, support vector machine; RF, random forest; CNN, convolution neural network; LR, logistic regression.

Jain *et al*. [79] presented an attention-channel-based DL model for the UNet that can recognize carotid plaques in images of the internal carotid artery (ICA) and the common carotid artery (CCA). The experiments include 970 ICA images from the United Kingdom, 379 CCA images from diabetic patients in Japan, and 300 CCA images from postmenopausal women in Hong Kong. This is an ethnically unbiased, multi-center, multi-ethnic research study on evaluating CVD/Stroke risk. The DL-based UNet model shows higher accuracy (98.32%) for plaque segmentation in the far walls of the arteries [85]. It has been demonstrated that cIMT and carotid plaque derived as image-based phenotypes using carotid ultrasound, when integrated with conventional CVD risk indicators [85, 86, 87], improved CVD risk prediction [91, 92, 93, 94, 95, 96].

## 4. Genomics-Based Biomarkers as Features for AI-Based CVD Diagnosis

Some studies have focused on incorporating multivariate biomarkers, leading to multivariable prediction models, to improve diagnosis and CVD risk stratification [97, 98]. Regarding *in vitro* biomarkers, the molecules can be isolated from the serum and/or plasma of asymptomatic subjects and CVD patients. The prediction models analyze the diverse circulating molecules, where these multivariate biomarkers represent the development of atherosclerosis and coronary arteries at various levels. Such GBBM includes cellular, biochemical, epigenetic, and transcriptional biomarkers towards the development of CVD and is discussed below. Further, Table 3 (Ref. [99, 100, 101, 102, 103, 104, 105, 106, 107, 108, 109, 110, 111, 112, 113, 114, 115, 116, 117, 118, 119, 120, 121, 122, 123, 124, 125, 126, 127, 128, 129, 130, 131, 132, 133, 134, 135]) summarizes the effect of GBBM on CVD.

**Table 3.** **Studies showing Genomics-based biomarkers (features) responsible for CVD, CHD, and HF**.

SN	Studies	Year	REF	Source	Biomarker Nomenclature	Observations	Clinical Outcome
Class 1: Cellular-based biomarkers (CBBM)							
1	Shantsila *et al*. [100]	2014	26	PBMC	CCR2-monocytes; CD14+CD16++; CD14++CD16+CCR2+	CHD patients had lower levels of CD14 and D14+CD16++CCR2-subpopulation expression.	Diagnosis
2	Weber *et al*. [103]	2016	108				
3	Williams *et al*. [102]	2021	104				
4	Arbel *et al*. [99]	2012	28	Leucocytes	The ratio of Neutrophils to Lymphocytes (N/L)	CHD severity and plaque vulnerability increase with an elevated (N/L) ratio.	Predictions and Diagnosis.
5	Teperman *et al*. [101]	2017	54				
6	Tareen *et al*. [125]	2022	21				
7	Berezin *et al*. [104]	2014	35	PBMC	Endothelial progenitor cells (EPCs)	Reduction in cell count and functional disability in CHD patients; linked to coronary lesion severity and sub-stent plaque burden.	Future CV events/PCI follow-up is diagnostic/predictive.
8	Otto *et al*. [126]	2017	44				
9	Kim *et al*. [105]	2014	28	Blood	CD31+, hs-CRP	Elevated CD31+ cells in unstable angina patients; links with atherosclerotic coronaries.	Predictions and Diagnosis of unstable angina.
10	Yuan *et al*. [106]	2020	34				
Class 2: Biochemical-based biomarkers (BCBM)							
SN	Studies	Year	REF	Source	Biomarker nomenclature	Observations	Clinical Outcome
1	Blankenberg *et al*. [111]	2001	19	Serum	sICAM-1/sVCAM-1	Significantly higher in unstable angina patients.	Predictions and Diagnosis of ACS.
2	Hulok *et al*. [110]	2014	16				
3	Yan *et al*. [107]	2021	113	Plasma	MCP-1	RCA identifies coronary atherosclerosis in UA patients; ACS patients have high concentrations.	Predicative increased risk of mortality or AMI.
4	Balın *et al*. [108]	2012	64	Serum	LOX-1	Higher levels in CHD patients with more severe disease.	Predictive and diagnosis of future CHD.
5	Sawamura *et al*. [109]	2015	85				
6	Hudzik *et al*. [115]	2014	26	Plasma	PTX3	Reduced level of PTX3 results in plaque vulnerability.	Diagnostic
7	Cavusoglu *et al*. [113]	2011	57	Serum	IL-10	Lowered in patients with ACS.	Predictive/diagnostic of long-term negative outcomes.
8	Kahles *et al*. [114]	2020	35				
9	Dechkhajorn *et al*. [112]	2020	49	Serum	IL-8	Increased levels in patients with CHD.	Predictive/diagnostic of long-term outcomes.
10	Ridker *et al*. [127]	2021	163	Serum	IL-6	High concentration among patients with multivessel atherosclerosis and calcified plaque, as measured by CCTA.	Diagnostic
11	Moore *et al.* [128]	2019	09				
Class 3: Epigenetic-based (Genetic) biomarkers (EpiBBM)							
SN	Studies	Year	REF	Source	Biomarker Nomenclature	Observations	Clinical Outcome
1	Lopes *et al*. [116]	2019	39	Lymphocytes	LINE-1	Lower CHD methylation	Identifies or predicts a higher risk of acute events and fatality.
2	Kim *et al*. [117]	2010	26	Lymphocytes	Alu/Sat2	Higher CHD methylation	Diagnostic
3	Li *et al*. [129]	2021	43	Lymphocytes	PLA2G7	Significant promoter methylation in CHD	Gender and age-specific diagnostic/predictive of CHD risk.
4	Wang *et al*. [130]	2022	265	Lymphocytes	ABCA1	Higher mutagenesis in CHD patients linked to low HDL; ageing, CHD in men.	Diagnostic
5	Gilham *et al*. [131]	2016	63	Plasma/Serum	Microarray	MiR-17-92 cluster downregulation, miR-126, miR-145, miR-155 upregulation, and miR-133 and miR-208a upregulation are all linked to CHD severity.	Diagnostic
6	Larsen *et al*. [132]	2021	55				
7	Gallo *et al*. [118]	2021	67	Serum	mir-197/mir-223	Patients with CHD have elevated levels.	Diagnostic
8	Doroschuk *et al*. [119]	2021	59	Plasma	Realtime PCR	High amounts of miR-17-5p are linked to the severity of CHD.	Diagnostic
9	Zhao *et al*. [133]	2017	43	Plasma	mir-214	Concentrations in the bloodstream that correlate with the degree of coronary stenosis.	Diagnostic
10	Hu *et al*. [120]	2022	43				
Class 4: Transcriptional-based biomarkers (TBBM)							
SN	Studies	Year	REF	Source	Biomarker Nomenclature	Observations	Clinical Outcome
1	Infante *et al*. [124]	2017	162	Leucocytes	Homer1/IL-1β/TNF-α	CHD patients have higher mRNA levels than healthy controls.	Diagnostic
2	Holvoet *et al*. [134]	2016	34	Monocytes	MT-COI	Low levels associated with CHD.	Predictive events related to CHD.
3	Yan *et al*. [135]	2014	71	PBMCs	MSH2/XRCC1/ATM	Increased upregulation in diabetic CHD patients.	Diagnostic
4	Yang *et al*. [123]	2020	36	PBMCs	Myocardin/GATA4/Nkx2.5	Higher levels of transcription in patients correlate with disease severity.	Diagnostic
5	Frambach *et al*. [121]	2020	106	Monocytes	Microarray	ABCA1, ABCG1, and RGS1 are suppressed, but ADRB2 and FOLR3 are increased.	Diagnostic
6	Fan *et al*. [122]	2021	44	PBMCs	Microarray	Upregulation of EGR1 levels can differentiate ischemia from non-ischemic CHD patients.	Diagnostic

CVD, cardiovascular disease; CBBM, cellular-based biomarkers; SN, serial number; REF, reference; N/L, neutrophils to lymphocytes; CHD, coronary heart disease; PBMC, peripheral blood mononuclear cells; HF, heart failure; AMI, acute myocardial infarction; UA, unstable angina; hs-CRP, high-sensitivity c-reactive protein; BCBM, biochemical-based biomarkers; EpiBBM, epigenetic-based biomarkers; EPC, endothelial progenitor cell; ACS, acute coronary syndrome; sICAM-1, soluble intercellular adhesion molecule-1; sVCAM-1, circulating vascular cell adhesion molecule-1; MCP-1, monocyte chemoattractant protein-1; RCA, right coronary artery; TBBM, transcriptional-based biomarkers; LOX-1, lectin-like oxidized low-density lipoprotein receptor 1; PTX3, pentraxin 3; IL, interleukin; CCTA, coronary computed tomography angiography; LINE-1, long interspersed nuclear elements-1; ABCA1, ATP-binding cassette transporter A1; mir, micro RNA; IL-1β, interleukin-1 beta; TNF-α, tumor necrosis factor-alpha; MT-COI, mtDNA encoded cytochrome c oxidase subunit I; MSH2, MutS homolog 2; XRCC1, X-ray repair cross-complementing protein 1; ATM, ataxia telangiectasia; RGS1, regulator of G-protein-1; ADRB2, beta-2 adrenergic receptor; CV, cardio vascular; PCI, percutaneous coronary intervention; CCR2, C-C chemokine receptor type 2; FOLR3, folate receptor gamma; PCR, polymerase chain reaction; ABCG1, ATP-binding cassette protein G1; EGR-1, early growth response protein 1; HDL, hybrid deep learning.

### 4.1 Cellular-Based Biomarkers

In the progression of CVD, circulating cells produce a broad spectrum of biomarkers [136]. This reveals that atherosclerosis and cardiovascular risk factors increase monocytes [137]. Monocyte subpopulations with various surface markers, functional changes, and gene expression alterations play diverse roles in atherogenesis [138]. It has been shown that serum leukocyte concentration and neutrophil/lymphocyte ratio predict plaque susceptibility [99, 100]. Several studies show a correlation between CVD risk factors, coronary lesion severity, and functional impairment [99, 101, 102, 103, 139]. Flow cytometry has revealed a link between the number of CD31 (+) cells and the density of atherosclerotic arteries [104, 140]. Kim *et al*. [105] show the molecular markers to monitor the CD31(+) cell activity in the blood of CHD patients. It reveals a strong link between the number of CD31(+) cells that trigger atherosclerosis [103, 106].

### 4.2 Biochemical-Based Biomarkers

Inflammatory biomarkers may be beneficial in diagnosing healthy individuals for CVD risk [141]. Several biomarkers have been identified recently, although none have been linked to imaging characteristics. Transforming growth factor beta 1 (TGF-β1) [142], cellular adhesion molecules (CAM) [143], monocyte chemoattractant protein-1 (MCP-1) [107], stromal cell-derived factor-1 (SDF-1) [108], lectin-like oxidized low-density lipoprotein receptor 1 (LOX-1) [109], haemoglobin A1c (HbA1c) [110, 111, 112], interleukin (IL) [113, 114], and pentraxin 3 (PTX3) [115] are strongly associated with the development of CVD in patients.

### 4.3 Epigenetic-Based Biomarkers

Epigenetic changes are important in CVD and atherosclerosis [116, 117]. Deoxyribonucleic acid (DNA) methylation, histone changes, and non-coding RNA (ncRNA) regulate epigenetic pathways [118]. Several studies evaluated the methylation proportion of genomic DNA from blood cells [144]. There is a strong relationship between the DNA methylation process and CVD or acute coronary syndrome (ACS) [145, 146]. A methylation pattern and a methylation signature can be used as predictive biomarkers for increased cardiac events, ischemic heart disease, stroke, and patient mortality [119]. Gallo *et al*. [118] proposed a plasma MiR-17-92 cluster downregulation, miR-126, miR-145, miR-133, miR-208a, and miR-155 upregulation, linking these to CHD severity. Hu *et al. * [120] explained the role of plasma miR-214 concentrations in the bloodstream that correlated with the degree of coronary stenosis.

### 4.4 Transcriptional-Based Biomarkers

**Fig. 3.**
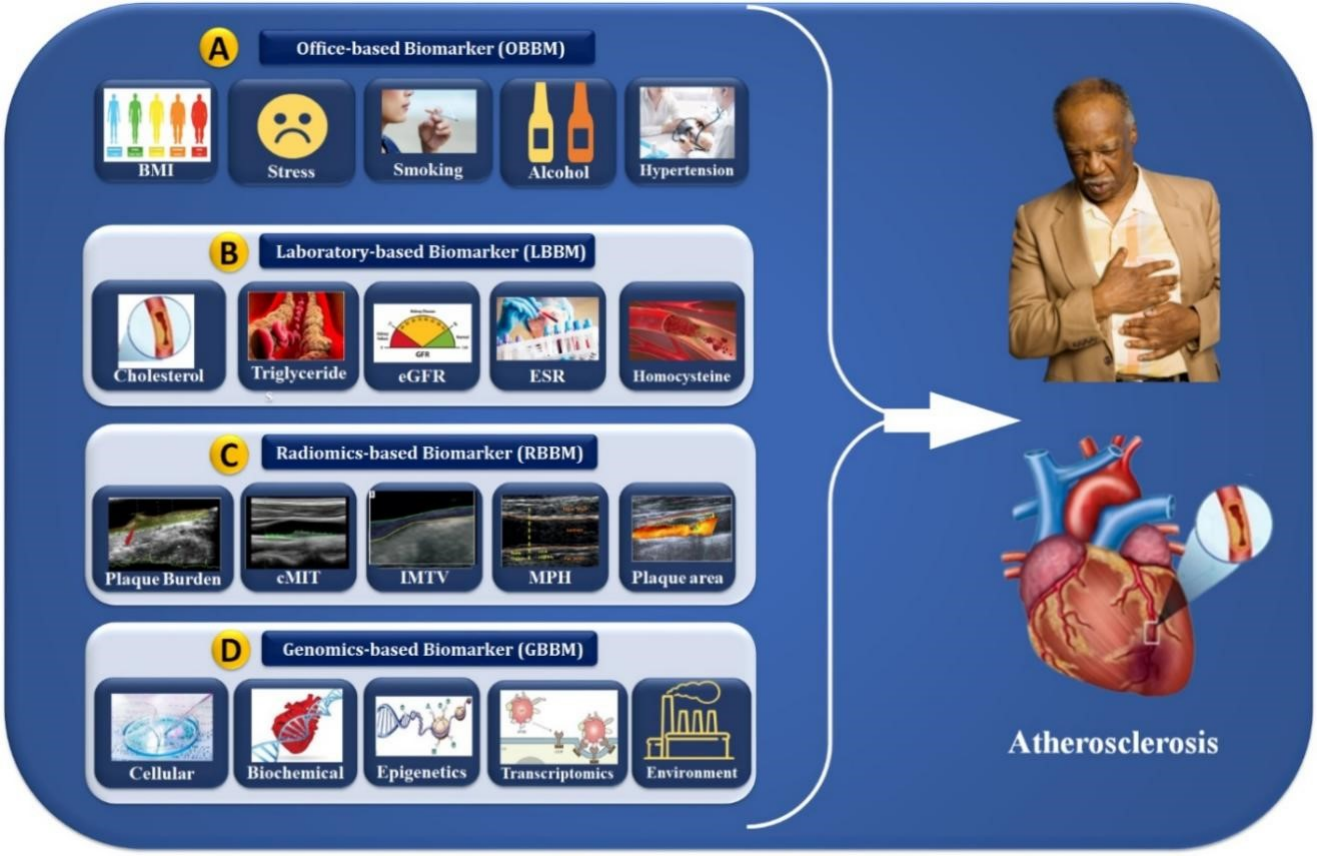
**Cardiac multivariate biomarker assessments (OBBM, LBBM, RBBM, and GBBM) for the risk stratification of atherosclerosis disease**. OBBM, office-based biomarkers; LBBM, laboratory-based biomarkers; RBBM, radiomics-based biomarkers; GBBM, genomics-based biomarkers; PBBM, proteomics-based biomarkers; EBBM, environment-based biomarkers; BMI, body mass index; eGFR, estimated glomerular filtration rate; ESR, erythrocyte sedimentation rate; cIMT, carotid intima-media thickness; IMTV, intima media thickness variability; MPH, maximum plaque height.

Genome-wide transcriptomic analysis has identified new disease biomarkers [120, 121]. Multiple investigations on blood cell profiling of gene expression have shown distinct transcriptional signatures in CVD patients and healthy participants [122, 147]. Yang *et al*. [123] showed that the transcription biomarkers named myocardin/GATA4/Nkx2.5 have higher levels in patients correlated with CVD disease severity. The upregulation of microarray EGR1 levels can easily differentiate ischemia from non-ischemic CHD patients [122]. The expression pattern correlated with CHD severity and gene function in vascular tissues demonstrated the synchronization between circulating cells and the atherosclerotic artery wall; for better identification and CVD risk prediction, one needs superior genomic biomarkers [148, 149]. Gene expression alteration may serve as biomarkers for disease development, progression, therapy efficacy, and environmental moderator effects. Specifically, 365 genes were discovered to be expressed differently between CHD patients and healthy participants [124, 150]. The carotid artery is a surrogate biomarker of coronary atherosclerosis when integrating cost-effective carotid B-mode ultrasound (cBUS) imaging techniques, and GBBM can lead to precise CVD risk stratification. However, the system becomes non-linear due to the presence of multiple covariates. AI plays an important role in reducing nonlinearity between covariates and outcomes. The following section discusses the role of AI in CVD risk stratification using the radiogenomics framework. The role of OBBM, LBBM, RBBM, GBBM, and EBBM is shown in Fig. 3. Previously, blood biomarkers and carotid ultrasonography have been used to predict the 10-year risk to improve plaque identification for monitoring atherosclerotic disease [151].

## 5. UltraAIGenomics: ${\mathrm{aiP}}^{3}$-Based Deep Learning for CVD Risk Stratification

Advances in machine learning (ML) and DL have been well-recognized in medical imaging [152, 153, 154, 155]. Deep neural networks (DNNs), a DL subgroup and work like a human brain, are considered a DL core [156, 157, 158]. Recent studies have used AI to risk stratify CVD in the RBBM [9, 10, 11, 159, 160, 161, 162, 163] and GBBM [27, 30, 164] frameworks. DL is becoming more popular because it (i) extracts the features automatically [165], (ii) can fuse with ML configurations for classification [157, 166], (iii) leverages UNet, and hybrid UNet-based DL strategies for segmentation [29, 38], and (iv) finally, it gives more accurate segmentation and solo or ensemble-based classification due to its ability to undergo forward and backward propagation by reducing different kinds of loss functions [79].

**Fig. 4.**
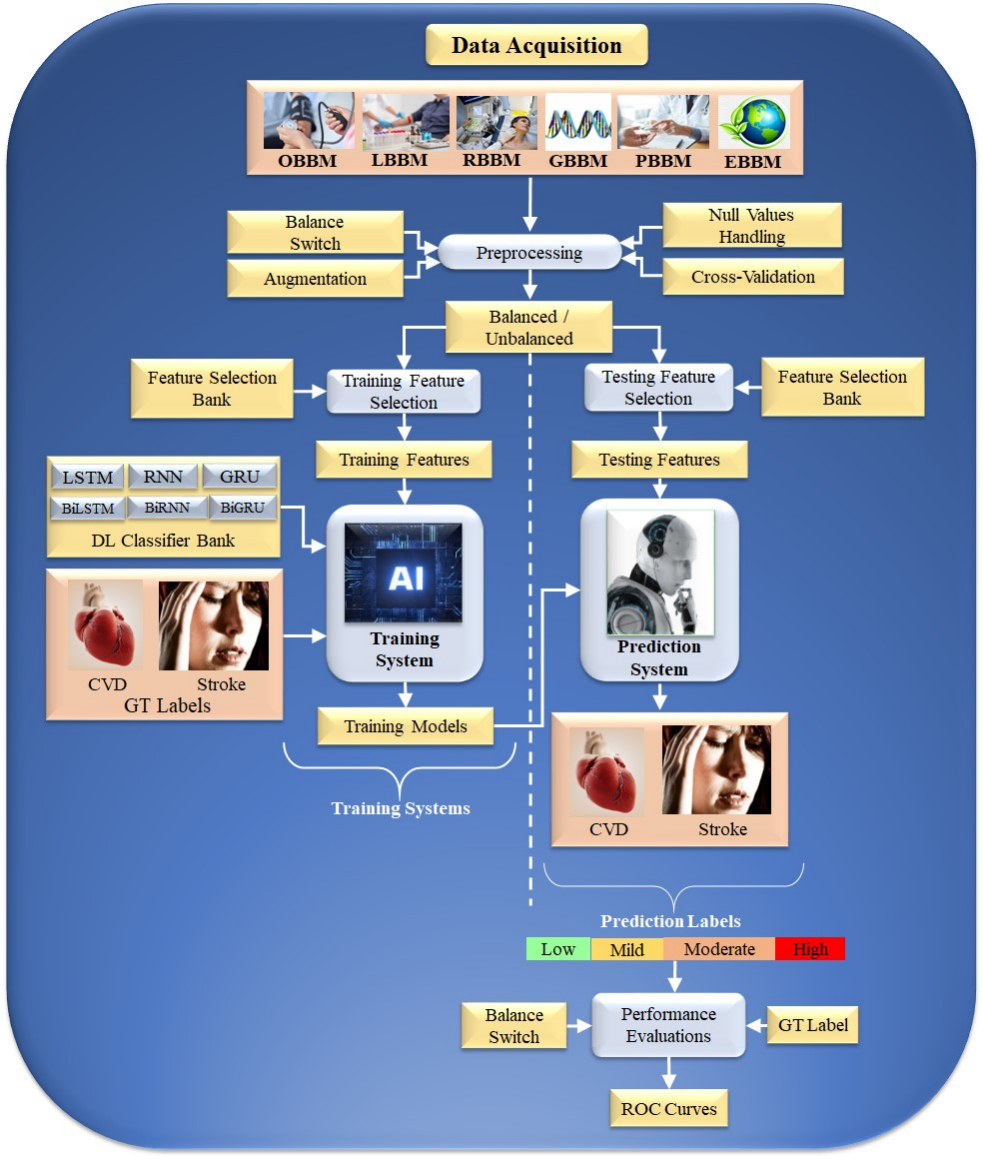
**DL-based architecture for CVD risk assessment**. OBBM, office-based biomarkers; LBBM, laboratory-based biomarkers; RBBM, radiomics-based biomarkers; GBBM, genomics-based biomarkers; PBBM, proteomics-based biomarkers; EBBM, environment-based biomarkers; LSTM, long short-term memory network; RNN, recurrent neural network; GRU, gated recurrent units; Bi, bidirectional; CVD, cardiovascular disease; DL, deep learning; GT, ground truth; AI, artificial intelligence; ROC, receiver operating characteristic.

Typical Deep Learning paradigm for CVD risk stratification: An Overall system DL is an effective strategy because it uses the underlying knowledge base to create automated features and offers a better training paradigm due to a profound number of NN layers that adjust the nonlinearity among both variables (covariates) and the gold standard. Fig. 4 depicts a typical DL system. The input acquisition consists of several biomarkers, namely, OBBM, LBBM, carotid image-based phenotypes (CUSIP) under the class of RBBM, medication utilization (MedUSE), GBBM, PBBM, and EBBM.

### 5.1 Training and Prediction

The architecture consists of two halves. The left and right half is the training subsystem, and the right is the prediction subsystem. The DL training classifiers consist of one of the DL classifiers, namely, long short-term memory network (LSTM), recurrent neural network (RNN), gated recurrent units (GRU), bidirectional LSMT (BiLSTM), bidirectional RNN (BiRNN), and bidirectional GRU (BiGRU) (presented in the following subsection). Along with the DL classifier bank, there are supervised clinical risk labels representing ground truth (GT), such as heart failure (or high CVD risk) and stroke [159, 167]. This GT representing the CAD includes computed tomography (CT) coronary score [168] or quantification of CAD lesions using intravascular ultrasound (IVUS) [169, 170]. Several non-linear training-based approaches have been shown in heart disease risk stratification [10, 160, 163, 171].

#### Deep Learning Classifier Banks

The RNN [172], BiRNN [173], LSTM [174], BiLSTM [175], GRU [176], and BiGRU [177] models evaluate sequential data, such as electrocardiograph (ECG) [176, 178], text [174], speech [179], localization of myocardial infraction [175] and handwriting [180, 181]. These models contain a set of continuous data patterns.

### 5.2 Radiomics-Based Biomarkers: DL-Based Plaque Wall Segmentation and CUSIP Measurement 

**Fig. 5.**
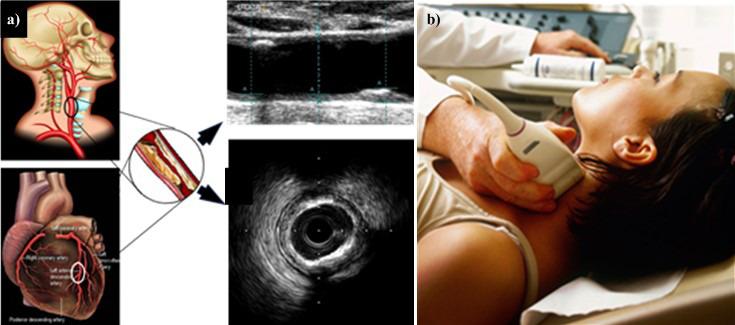
**CUSIP Measurement**. (a) Carotid artery is a potential surrogate marker for the coronary artery. Also, the grayscale images are shown for carotid longitudinal B-mode US scans and coronary IVUS transverse scan (b) B-mode carotid longitudinal imaging system using linear ultrasound [43]. CUSIP, carotid image-based phenotypes; US, Ultrasound; IVUS, intra vascular ultrasound.

CUSIP refers to image-based carotid artery phenotypes [63, 68, 182, 183]. This training program is adaptable to non-linear adaptation [10, 160, 163, 171, 184, 185]. Fig. 5 (Ref. [43]) represents the cBUS scan and its corresponding coronary atherosclerotic disease.

The DL system can be used to measure plaque burden, plaque area, average and maximum cIMT, IMTV, geometric and morphological total plaque area (TPA), and stenosis/lumen diameter [186, 187, 188]. This DL system segments the walls and then computes CUSIP [189, 190]. The supervised DL-based CVD risk stratification uses the GT for training and performance evaluation.

### 5.3 Plaque Wall Segmentation in the UNet-Based Deep Learning Framework

Jain *et al*. [29] proposed a U-shaped network (UNet) model for detecting atherosclerotic plaque. The model uses four layers of DL and a pair of encoders and decoders. Utilizing the capabilities of automated feature extraction and reconstruction of desired forms, UNet-based DL has recently overtaken the medical image segmentation market of imaging modalities [191].

**Fig. 6.**
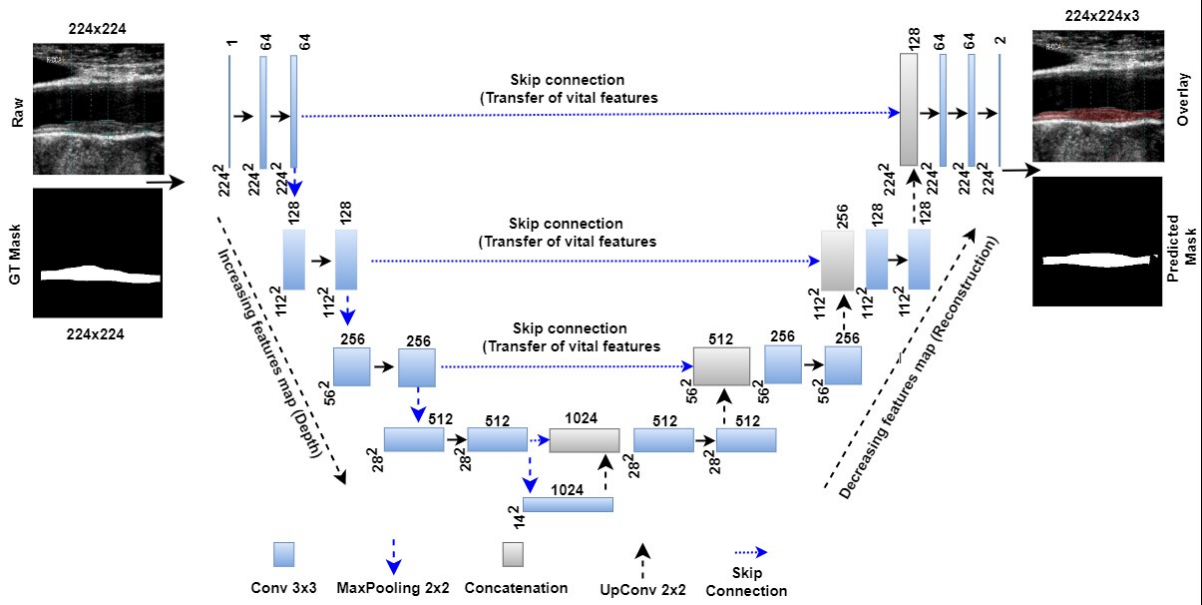
**UNet model for segmentation of the atherosclerotic plaque wall [29]**. GT is ground truth, and Conv is convolution. The UNet-based DL model can transmit features extracted from the encoder to the decoder phases and preserve the desired features during shape reconstruction at the decoder phase. In contrast to geometric curves based on level sets, UNet-based DL does not require the positioning of the first curves. Moreover, it needs the gold standard for training the UNet-based DL models [193, 194]. DL, deep learning; GT, ground truth; UNet, U-shaped network.

Ronneberger *et al*. [192] first announced UNet as an image segmentation method for comparison with conventional standard segmentation techniques in 2015. The architecture of this UNet is depicted in Fig. 6 (Ref. [29, 193, 194]), showing the bridge network, encoders, decoders, skip connections, loss function conditions, and binary conversion (so-called “softmax layer”) are primary components of UNet architecture. When coupled with the ability to select the highest-level characteristics called max pooling, this historical breakthrough of down and up convolution boosts the automated feature extraction process [192].

### 5.4 Long Short-Term Memory Classifier

**Fig. 7.**
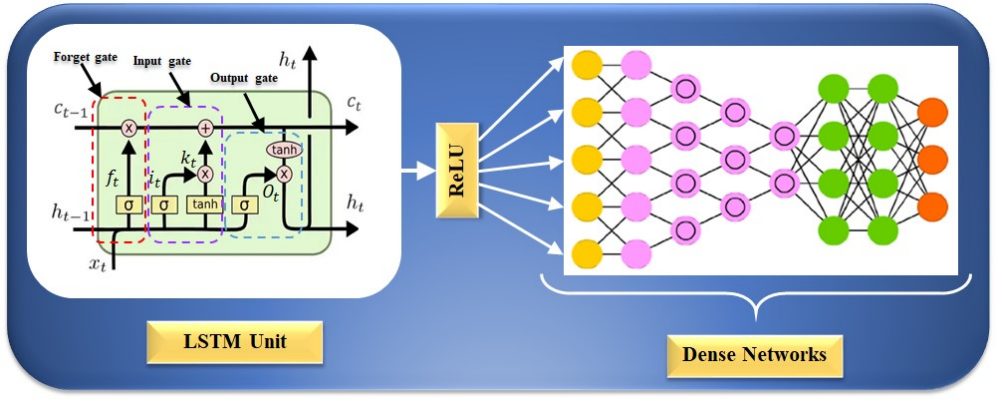
**LSTM architecture for CVD risk stratification**. LSTM is a long short-term memory network, and ReLU is a rectified linear unit. CVD, cardiovascular disease; LSTM, long short-term memory network.

The RNN model cannot work to learn long-term dependencies, which results in a bridge problem when connecting old and new data [195, 196]. This seldom causes the vanishing gradient problem, in which error signals vanish after backpropagation, leading to challenges in the model design [179]. LSTM networks replace the hidden layer node with a memory unit to improve the RNN model [197]. The cell’s state is the master key to archiving past data. There are three gate architectures for using the sigmoid activation function and the point-by-point product operation to modify or remove data from the current state of the cell [197]. The internal structure of an LSTM unit is depicted in Fig. 7; the *forget gate*, *input gate*, and *output gate* can be seen from left to right. An LSTM network could process sequence information in the cumulative linear form to avoid gradient vanishing and learn long-period information. The LSTM can be trained to understand data over extended periods. The equation for the *forget gate* is given as follows:

(1) $$f_{t}=\sigma \,\left(\omega_{f}*\left[h_{t-1},x_{t}\right]+b_{f}\right)$$

whereas, $f_{t}$ is the output value of the *forget gate* and $h_{t-1,}$ is the output value for the preceding state, $x_{t}$ is input value present state, $\omega_{f}$ is a weight matrix, σ is the sigmoid activation function, and $b_{f}$ represents bias vector. The equation for the *input gate* is given as:

(2) $$i_{t}=\sigma \,\left(\omega_{i}*\left[h_{t-1},x_{t}\right]+b_{i}\right)$$

(3) $$k_{t}=\tanh\left(\omega_{k}*\left[h_{t-1},x_{t}\right]+b_{k}\right)$$

whereas $i_{t}$ and $k_{t}$ are outputs of the *input gate*, $\omega_{k}$ and $b_{k}$ are the weight matrix and bias vectors, and *tanh* is the activation function of the *input gate.* The equation for the *output gate* is as follows,

(4) $$O_{t}=\sigma \,\left(\omega_{o}*\left[h_{t-1},x_{t}\right]+b_{o}\right)$$

(5) $$h_{t}=O_{t}\,\sigma *\tanh\left(C_{t}\right)$$

where $O_{t}$ is the output value of the *output gate*, $\omega_{o}$ and $b_{o}$ are the weight matrix and bias vector of the *output gate*’s, σ is the sigmoid activation function, and $h_{t}$ indicates the current output value of the present state. Now, the revised state cell is,

(6) $$C_{t}=f_{t}*C_{t-1}+i_{t}*k_{t}$$

whereas $C_{t}$ represents the state of the cell at the current moment and $C_{t-1}$ represents the state of the cell at the prior instant.

#### Factors Affecting DL Architecture and its Optimization

The challenge with DL solutions is that they need optimization during training using hyperparameters [38, 80]. DL-based training requires several epochs, the best learning rate, batch size, batch normalization, and dropout layers to avoid overfitting or generalization without memorization [198, 199]. Further, the patients with CVD risk with other comorbidities cause the dynamics to be non-linear between covariates and the gold standard [200]. Thus, to get the best DL architecture, one needs an extensive data framework with several different diagnostic sources and multiple data sets [201].

## 6. Explainability, Pruning, Bias, and Miscellaneous Factors Affecting CVD

### 6.1 The Role of Artificial Intelligence Explainability

Explainability is critical to CVD risk assessment because it gives medical professionals and physicians insight into the underlying characteristics and circumstances that influence AI models’ predictions. The most crucial part of AI or deep learning is understanding how AI’s “black box” works. Medical professionals are more likely to understand the “black box” if the results can be interpreted and questioned [202]. Explainability breaks down the “black-box” aspect of complicated deep learning (DL) models, allowing physicians to pinpoint the precise genetic or imaging characteristics that have the most significant impact on the model’s risk predictions. With a more detailed understanding of the illness processes and risk variables made possible by this information, doctors are better equipped to decide how best to treat patients and implement intervention measures. Explainability also encourages cooperation between AI systems and human professionals, enabling a mutually beneficial partnership in which AI enhances clinical decision-making rather than replacing it. Since the AI model may shed light on complex disorders using tools like local interpretable model-agnostic explanations (LIME) and shapley additive explanations (SHAP), it has gained credibility among medical professionals [154, 203]. Like other lesions, carotid lesions can be displayed using GradCAM, GradCAM+, or GradCAM++ [204]. This opens the door for a wider acceptance of AI models in the medical field. As a result, AI devices can be improved and made economic if they can be explained [205].

### 6.2 The Role of Pruning-Based Deep Learning Systems

Edge devices are becoming increasingly important as cloud-based systems and the internet improve [206]. Edge devices are extremely important when using trained AI models for future predictions or disease risk stratifications in mobile frameworks [207]. There is a requirement to deploy compressed models since huge data models cannot be deployed on edge devices [208]. Image-based deep learning models such as fully convolutional networks (FCN) or segmentation networks (SegNet) [36] can be pruned using evolutionary algorithms such as particle swarm optimization (PSO), genetic algorithms (GA), wolf optimization (WO), and differential evolution (DE) [209]. The future of radiomics-based CVD risk stratification fused genetic-based paradigms can be compressed and deployed on edge devices for rural areas, especially in third-world nations [210].

### 6.3 Role of Bias in Artificial Intelligence

Evaluating bias in AI models has gained much greater significance in recent years [211, 212]. Earlier computer-aided diagnosis techniques showed a lack of bias in evaluations [200]. To reduce bias, a large sample size, appropriate clinical testing, incorporating comorbidities, using big data configurations, using unseen data analysis, and the scientific validation of training model design are all strategies that can be utilized [34, 168]. Important phases in patient risk stratification include determining the AI RoB [34, 35, 213] and suitably modifying diagnostics and treatment.

## 7. CVD Risk Calculators: Conventional vs. AI-Based and its Practical Implications

Researchers developed five generations of cardiovascular risk stratification methods over time. The first generation used manual calculations, assessing risk based on blood tests, family history, and carotid ultrasound [214]. The second generation employed calculators like framingham risk score (FRS) and atherosclerotic cardiovascular disease (ASCVD) but had variability [26]. The third generation introduced image-based strategies using AtheroEdge™ systems for automation [29]. The fourth generation used machine learning, collecting data from MRI, US, and CT with automated segmentation and classifiers like SVM and random forest (RF). In the fifth generation, deep learning was employed for detailed multiclass risk assessment, representing a comprehensive evolution from manual calculations to advanced DL-based approaches with the potential for monitoring treatment responses [214].

### Practical Implications of the Proposed AI Model 

The AtheroEdge™ 3.0 classification system, powered by ML and DL, has practical implications. It offers precise risk stratification for diabetes using biomarkers like OBBM, LBBM, and RBBM, classifying them into low, moderate, and high-risk categories with over 26 models [215]. It is adaptable for various applications by incorporating image-derived risk factors through AI-based radiomics analysis of carotid ultrasound images, using CNN, UNet, UNet+, SVM, RF, and logistic regression (LR) algorithms for high accuracy [204]. It is reliable for CVD risk assessment, handles large cohorts, and extends to oncology for cancer risk stratification [166].

AtheroEdge™ 3.0’s capability to assess the impact of additional features on classification performance is notable, making it a superior choice [216]. It evaluates models using metrics like accuracy, area under the curve (AUC), *p*-value, sensitivity, specificity, F1-score, mathew correlation coefficient, precision, and recall, aiding in model selection for specific applications [217].

## 8. Critical Discussions

The DL system needs to overcome key concerns like bias, explainability, ergonomic design, and affordability to ensure the safety and effectiveness of the medical product, such as CVD risk stratification.

### 8.1 Principal Findings

This is the first study of its kind (a) that combines radiomics and genomic biomarkers to detect the severity of CVD and stroke risk precisely and (b) that introduces a proposed ${\mathrm{aiP}}^{3}$ risk model based on a preventive, predictive, and personalized approach that uses DL to classify CVD and stroke risk more accurately. Using these two hypotheses, we demonstrated that CVD and stroke risk severity could be determined using RBBM and GBBM biomarkers in the DL framework. Such models can be considered personalized medicine frameworks [218]. One of the major innovations is to ensure that cBUS imaging and CVD genomic biomarkers are jointly used in the DL framework for CVD risk stratification provided for accurate, robust, real-time CVD risk assessment using combined RBBM and GBBM [219].

Platelet count, mean platelet volume (MPV), platelet RNA, and protein are all parameters that are used to evaluate platelet function and activity [220]. Platelets are small, anucleate cells that play a critical role in hemostasis and thrombosis, and abnormalities in their function have been implicated in various CVDs [221]. High platelet counts, increased MPV, and elevated levels of platelet RNA and protein have been associated with an increased risk of CVD and adverse cardiovascular events [148, 150]. Complete blood count (CBC) blood indices, including red blood cell (RBC) count, haemoglobin (Hb) concentration, hematocrit (Hct), mean corpuscular volume (MCV) and mean corpuscular haemoglobin concentration (MCHC), are routinely used to assess blood cell counts and morphology [222]. Abnormalities in these indices have been linked to various CVDs, such as anaemia, ischemic heart disease, and stroke [223]. The neutrophil to lymphocyte (N/L) ratio measures the balance between innate and adaptive immunity and has been proposed as a biomarker of inflammation and oxidative stress [224]. Elevated N/L ratios have been associated with an increased risk of CVD and adverse cardiovascular events and are thought to reflect chronic low-grade inflammation and impaired immune function [225]. In summary, platelet count, MPV, platelet RNA, protein, CBC blood indices, and N/L ratios are all parameters used to evaluate various aspects of cardiovascular health and disease [226]. Abnormalities in these parameters have been linked to increased risk of CVD and adverse cardiovascular events [227].

### 8.2 Benchmarking against Previous UltraGenomics-Based Systems

The benchmarking studies outlined in Table 4 (Ref. [9, 10, 11, 24, 29, 33, 62, 83, 228, 229, 230, 231, 232, 233, 234, 235, 236, 237]), consist of 17 attributes that are identified by the letter ‘K’ followed by a number. The first attribute, K0, refers to the serial number assigned to each study. The second attribute, K1, represents the name of the studies, while K2 represents the year of publication. The third attribute, K3, indicates the references used in the studies. The remaining 14 attributes, K4 through K17, are related to using different types of AI studies in CVD risk prediction. K4 through K9 represent six different types of AI-based biomarkers for CVD, including office-based blood biomarkers (OBBM), laboratory-based blood biomarkers (LBBM), radiology-based biomarkers (RBBM), genetic-based biomarkers (GBBM), proteomics-based biomarkers (PBBM), and environmental-based biomarkers (EBBM). Krittanawong *et al*. [228] elaborate on the rapid growth of digital technology adoption within healthcare, anticipating substantial improvements in care quality and global healthcare accessibility. However, they emphasize the necessity for more comprehensive data, efficacy studies, and objective outcomes to solidify the role of digital health in patient care. Another study by Jamthikar *et al*. [62] utilized ML techniques to stratify CVD risk in patients. This research underscores two of three pathways directly affecting atherosclerosis and highlights the superior performance of carotid ultrasound image-based calculators over standard methods. CVD risk stratification in patients using AI-based approaches is increasingly prevalent. Conversely, Saba *et al*. [229] offer a concise overview of the development of carotid atherosclerosis via B-mode ultrasound imaging. Their work underscores the inadequacies of conventional risk scores and explores the potential of machine learning-based tissue analysis to address these gaps. Gruson *et al.* [230] provide a comprehensive review of AI applications in genomics and imaging, noting the limited clinical implementation of several techniques. They anticipate that recent advancements in DL will revolutionize this domain, enhancing patient care in conjunction with human interpretation and clinical reasoning.

**Table 4.** **Benchmarking table for CVD risk using multivariate biomarkers**.

K0	K1	K2	K3	K4	K5	K6	K7	K8	K9	K10	K11	K12	K13	K14	K15	K16	K17
1	Krittanawong *et al*. [228]	2018	31	✓	✓	×	✓	✓	×	✓	✓	✓	NR	×	×	×	×
2	Arena *et al*. [232]	2018	202	✓	✓	×	✓	×	×	✓	×	✓	NR	×	×	×	×
3	Krittanawong *et al*. [234]	2017	88	✓	✓	×	✓	×	×	✓	×	✓	DL	×	×	×	×
4	Jamthika *et al.* [62]	2019	110	✓	✓	✓	×	×	×	✓	✓	×	HDL	×	×	×	×
5	Khanna *et al*. [24]	2019	54	✓	✓	✓	×	×	×	✓	✓	×	ML	×	×	✓	×
6	Saba *et al.* [229]	2021	125	✓	✓	✓	×	×	×	✓	✓	×	DL	×	×	×	×
7	Dainis *et al*. [33]	2018	83	✓	✓	×	✓	×	×	✓	×	✓	DL	×	×	×	×
8	Jamthikar *et al.* [9]	2020	40	✓	✓	✓	×	×	×	✓	✓	×	ML	×	×	×	×
9	Gruson *et al*. [230]	2020	42	✓	✓	×	✓	✓	×	✓	×	✓	HDL	×	×	×	×
10	Jamthikar *et al.* [11]	2020	118	✓	✓	✓	×	×	×	✓	✓	×	ML	×	×	×	×
11	Alimadadi *et al*. [231]	2020	56	✓	✓	×	✓	×	×	✓	×	✓	ML	×	×	×	×
12	Saba *et al.* [233]	2021	69	✓	✓	✓	×	×	×	✓	✓	×	ML	×	×	✓	×
13	Jamthikar *et al.* [10]	2021	85	✓	✓	✓	×	×	×	✓	✓	×	ML	×	×	✓	×
14	Westerlund *et al*. [235]	2021	167	✓	✓	×	✓	×	×	✓	×	✓	DL	×	×	×	×
15	Schiano *et al*. [236]	2021	29	✓	✓	×	✓	✓	×	✓	×	✓	ML	×	×	×	×
16	Jain *et al.* [29]	2021	67	✓	✓	✓	×	×	×	×	✓	×	ML	×	×	×	×
17	Staub *et al.* [237]	2010	25	✓	✓	✓	×	×	×	×	✓	×	NR	×	×	×	×
18	Jain *et al*. [83]	2022	85	✓	✓	✓	×	×	×	✓	✓	×	DL	×	×	✓	✓

K0, serial number; K1, studies; K2, year; K3, references; K4, OBBM; K5, LBBM; K6, RBBM; K7, GBBM; K8, PBBM; K9, EBBM; K10, preventive; K11, prediction; K12, personalized; K13, AI type; K14, FDA discussion; K15, clinical setting; K16, risk of bias; K17, AI explainability; CVD, cardiovascular disease; DL, deep learning; ML, machine learning; HDL, hybrid deep learning; NR, not reported; OBBM, office-based biomarkers; LBBM, laboratory-based biomarkers; RBBMM, radiomics-based biomarkers; GBBM, genomics-based biomarkers; PBBM, proteomics-based biomarkers; EBBM, environment-based biomarkers; AI, artificial intelligence; FDA, food drug administration.

In 2020, Song *et al*. [238] conducted a study involving 55 participants, concentrating on high-density lipoprotein (HDL) techniques for CVD risk stratification in patients. This investigation establishes a noteworthy correlation between carotid atherosclerotic image-based biomarkers, such as carotid intima-media thickness (cIMT) and plaque, and specific RA-associated inflammatory markers. They suggest integrating conventional image processing techniques, such as fast marching methods, for efficient segmentation of vascular plaque [136]. Alimadadi *et al*. [231] observe that integrating digital technologies into rheumatology healthcare is an emerging trend, offering a wide array of devices to facilitate personalized and continuous patient care. Gruson *et al*. [230] employ ML techniques for CVD risk stratification using a genomics approach, emphasizing their potential for preventive applications. However, none of the mentioned authors address the applicability of their methodologies for preventive and predictive purposes. Regrettably, the studies lack information on food drug administration (FDA) discussions, clinical contexts, risk of bias, and AI explainability [9, 11, 33, 83, 171, 228, 229, 230, 231, 232, 233, 238].

In contrast, our proposed study leverages 260 references and employs DL techniques for using Ultragenomics for CVD risk stratification. Our approach encompasses preventive, predictive, and personalized objectives, along with an explicit discussion of AI explainability during the FDA deliberations. However, details regarding the clinical setting and potential bias risk are absent.

### 8.3 Recommendations for Using the UltraAIGenomics Model for CVD/Stroke Risk

Following are guidelines for a proposed UltraAIGenomics model that can be used for CVD/Stroke risk stratification. The study proposes two hypotheses: (a) radiomics and genomic biomarkers have a strong correlation and can be used to detect the severity of CVD and stroke precisely, and (b) introduces a proposed (${\mathrm{aiP}}^{3}$) risk model that uses DL to classify CVD and stroke risk more accurately. We propose the following recommendations: (i) requires a clinical evaluation and scientific validation for reliable detection and CVD risk stratification, and (ii) requires hyper-parameter optimization in CVD/Stroke risk stratification. (iii) balancing the risk classes (control, low-risk, and high-risk) is the most effective way to minimize DL bias; (iv) with proper pruning and compression, DL systems can be adapted to edge devices; (v) a DL system that relies on surrogate carotid imaging can be cost-effective without compromising precision in CVD risk stratification.

### 8.4 Strengths, Weakness, and Extensions of the Study

This pilot review’s ability to risk stratify CVD and stroke patients by integrating RBBM and GBBM was a major strength. The first theory was supported by the biomarkers derived from radiological, biochemical, and morphological complexity that established a connection to CVD. A DL approach was presented to evaluate CVD and stroke risk by integrating RBBM and GBBM. While the system is quite straightforward, input data has always been a challenge since sample size leading to big data is required [239]. It requires optimization to eliminate the possibility of bias and generalization to account for comorbidities [240]. Further, carotid artery imaging must encounter all three segments, such as common, bulb, and internal [186], for best plaque measurements [68]. Better comprehensive feature space can be tried for superior DL-based classification [241, 242]. As part of extensions, conventional image processing can be fused with AI models for superior performance [243]. Ensemble-based solutions embedding with explainability for best feature selection followed by recurrent neural networks are possible extensions for superior CVD/Stroke risk solutions [244, 245].

### 8.5 Future Work

Nevertheless, it is imperative to recognize the constraints of our study. Notwithstanding the progress achieved, issues like AI ethics and design complexity remain major roadblocks that require attention. Furthermore, even though our research offers a strong framework for using genetic and radiomic biomarkers in CVD risk assessment, additional validation and improvement of the ${\mathrm{aiP}}^{3}$ model are required to guarantee its dependability and efficacy in various clinical contexts.

Future studies should concentrate on resolving these issues and expanding on our discoveries. Investigating the incorporation of cutting-edge technologies like Blockchain and IoMT into conventional healthcare procedures is one aspect of this, as is researching cutting-edge AI-driven strategies for improving explainability and lowering bias in CVD risk assessment models. Furthermore, research on the long-term clinical results and financial viability of using genetic and radiomic biomarkers in regular cardiovascular disease evaluation is necessary. We can keep advancing the area of cardiovascular medicine and eventually enhance patient care globally by embracing these new research directions.

## 9. Conclusions

The presented research has important theoretical and practical ramifications that have the potential to drastically alter how CVD is evaluated. First, we explored biomarkers like IL, CD31+, EPCs, and high-sensitivity c-reactive protein (hs-CRP), which strongly connect with CVD prognosis. High levels of CRP in people with low blood pressure and a recent heart attack history can predict future coronary events. Besides this, the radiomic features such as plaque burden, plaque area, and carotid intima thickness provide a quantified view of CVD risk. Second, we introduced the ${\mathrm{aiP}}^{3}$ risk model, a breakthrough in CVD and stroke risk assessment. This model uses DL to untangle the complex relationship between multiple biomarkers and outcomes. It emphasizes atherosclerosis’s genetic and radiomic markers in the carotid, coronary, and aortic arteries. DL helps us manage the complexity of these biomarker interactions. During our narrative review, we addressed important issues like AI bias, explainability, and pruning.

We proposed a cloud-based system design to balance precision and interpretability in CVD risk assessment, emphasizing the need for ethical and unbiased AI in clinical practice. Additionally, we touched on platelet function, complete blood count (CBC), and diagnostic methods, adding depth to CVD assessment. In conclusion, our narrative review lays a strong foundation for using genomic and radiomic biomarkers in precise CVD risk assessment. The ${\mathrm{aiP}}^{3}$ model, powered by DL, brings us closer to personalized and preventive cardiovascular health management. As we navigate the complexities of AI ethics and design, we pave the way for a future where technology enhances patient outcomes seamlessly.

## Abbreviations

ARDS, Acute respiratory distress syndrome; ASCVD, Atherosclerotic cardiovascular disease; ANS, Autonomic nervous system; AUC, Area-under-the-curve; AI, Artificial Intelligence; ACS, Acute coronary syndrome; BMI, Body mass index; CAD, Coronary artery disease; CAS, Coronary artery syndrome; CHD, Coronary heart disease; CT, Computed Tomography; CUSIP, Carotid ultrasound image phenotype; CV, Cross-validation; CVD, Cardiovascular disease; CVE, Cardiovascular events; CNN, Convolution neural network; DL, Deep learning; DM, Diabetes mellitus; DT, Decision tree; EC, Endothelial Cell; EBBM, Environment-based biomarkers; GT, Ground truth; GBBM, Genetically based biomarkers; HTN, Hypertension; HDL, Hybrid deep learning; ICAM, Intercellular adhesion molecule; VCAM, Vascular cell adhesion molecule; LBBM, Laboratory-based biomarker; LIME, Local Interpretable Model-Agnostic Explanations; MRI, Magnetic Resonance Imaging; NR, Not reported; NPV, Negative predictive value; NB, Naive Bayes; Non-ML, Non-machine learning; OBBM, Office-based biomarker; OH, Orthostatic hypotension; OxLDL, Oxidation of low-density lipoprotein; PE, Performance evaluation; PPV, Positive predictive value; PCA, Principal component analysis; PBBM, Proteomics based bio-markers; PRISMA, Preferred Reporting Items for Systematic Reviews and Meta-Analyses; PTC, Plaque tissue characterization; RA, Rheumatoid arthritis; RF, Random forest; ROS, Reactive Oxides Stress; RoB, Risk of bias; ROC, Receiver operating-characteristics; RNN, Recurrent neural network; SCORE, Systematic coronary risk evaluation; SMOTE, Synthetic minority over-sampling technique; SVM, Support vector machine; SHAP, Shapley Additive Explanations; TPA, Total plaque area; TC, Tissue Characterization; US, Ultrasound.

## Appendix

// Pseudo code for AI-based CVD Diagnosis using Radiomics-based Biomarkers

// Function to perform AI-based CVD diagnosis using radiomics-based biomarkers

Procedure PerformAIBasedCVDDiagnosis:

Input: BiomarkersList, ImagingMethodsList

Output: DiagnosisResult

// Initialize variables

CVDImagingData = LoadCVDImagingData(BiomarkersList, ImagingMethodsList)

// Display information about the loaded imaging data

DisplayImagingDataInfo(CVDImagingData)

// Extract features using stochastics-based methods (SBM)

SBMFeatures = ExtractFeaturesUsingSBM(CVDImagingData)

// Display the features extracted using SBM

DisplaySBMFeatures(SBMFeatures)

// Train and evaluate a stochastics-based model for CVD risk stratification

SBMModel = TrainAndEvaluateSBMModel(SBMFeatures)

// Display performance metrics of the SBM model

DisplaySBMModelPerformance(SBMModel)

// Extract features using deep learning (DL) based radiomics

DLFeatures = ExtractFeaturesUsingDLRadiomics(CVDImagingData)

// Display the features extracted using DL-based radiomics

DisplayDLFeatures(DLFeatures)

// Train and evaluate DL-based model for CVD risk stratification

DLModel = TrainAndEvaluateDLModel(DLFeatures)

// Display performance metrics of the DL model

DisplayDLModelPerformance(DLModel)

// Combine the results from SBM and DL models

CombinedResults = CombineSBMAndDLResults(SBMModel, DLModel)

// Display the final diagnosis results

DisplayDiagnosisResults(CombinedResults)

// Output the final diagnosis result

DiagnosisResult = GenerateDiagnosisReport(CombinedResults)

// Return the diagnosis result

Return DiagnosisResult

End Procedure

Methodology

The methodology outlined in the pseudo-code, describes an AI-based approach for CVD diagnosis using radiomics-based biomarkers. It involves three main steps: preprocessing, augmentation, and deep convolutional neural network (CNN) architecture. In the preprocessing phase, CVD imaging data is loaded, and features are extracted using two distinct techniques: DL-based radiomics and stochastics-based methods (SBM). If the data is in high-dimensional RNA sequences, it is first converted into 2D images [246]. The augmentation phase aims to increase the size of the dataset. Finally, a deep CNN architecture, which comprises the convolutional layers for feature extraction and fully connected layers for classification, is implemented. The final diagnosis is produced by combining the performance metrics of traditional stochastics-based methods and deep learning techniques, which are displayed. Combining the best features of SBM and DL methodologies improves the precision and consistency of CVD diagnosis. A method for visualizing the regions of an image that a deep learning model concentrates on when generating predictions is called gradient-weighted class activation mapping, or Grad-CAM. It facilitates the comprehension of the image’s most significant components for the model’s decision-making process.
